# Optimal antithrombotic therapy in ischemic stroke patients with non-valvular atrial fibrillation and atherothrombosis: study protocol for a randomized controlled trial

**DOI:** 10.3389/fneur.2024.1468523

**Published:** 2024-10-23

**Authors:** Shuhei Okazaki, Haruko Yamamoto, Koko Asakura, Katsuhiro Omae, Hirotada Maeda, Kanta Tanaka, Shiro Yamamoto, Teruyuki Hirano, Yasuyuki Iguchi, Manabu Sakaguchi, Masatoshi Koga, Masafumi Ihara, Kazunori Toyoda, Teruo Noguchi, Nobuyuki Sakai, Hiroshi Yamagami

**Affiliations:** ^1^Department of Neurology, NHO Osaka National Hospital, Osaka, Japan; ^2^Department of Neurology, Osaka University Graduate School of Medicine, Osaka, Japan; ^3^Department of Data Science, National Cerebral and Cardiovascular Center, Osaka, Japan; ^4^Department of Cerebrovascular Medicine, National Cerebral and Cardiovascular Center, Osaka, Japan; ^5^Department of Stroke and Cerebrovascular Medicine, Kyorin University School of Medicine, Tokyo, Japan; ^6^Department of Neurology, The Jikei University School of Medicine, Tokyo, Japan; ^7^Department of Neurology, Osaka General Medical Center, Osaka, Japan; ^8^Department of Neurology, National Cerebral and Cardiovascular Center, Osaka, Japan; ^9^Department of Cardiovascular Medicine, National Cerebral and Cardiovascular Center, Osaka, Japan; ^10^Department of Neurosurgery, Seijinkai Shimizu Hospital, Kyoto, Japan; ^11^Division of Stroke Prevention and Treatment, Institute of Medicine, University of Tsukuba, Ibaraki, Japan

**Keywords:** antiplatelet, anticoagulant, nonvalvular atrial fibrillation (NVAF), atherothrombosis, ischemic stroke, randomized controlled trial

## Abstract

**Background:**

The addition of antiplatelet therapy to anticoagulant therapy in patients with stroke with non-valvular atrial fibrillation (NVAF) and atherothrombotic disease may increase bleeding risk without reducing recurrent stroke risk.

**Aims:**

To evaluate the clinical benefits of anticoagulant monotherapy compared to combination therapy with anticoagulants and antiplatelet agents.

**Methods and design:**

This is an investigator-initiated prospective multicenter, randomized, open-label, parallel-group clinical trial. Patients with NVAF and atherothrombotic disease who have had a recent ischemic stroke or transient ischemic attack will be eligible to participate in this trial.

**Study outcomes:**

The primary outcome is a composite of ischemic cardiovascular events, including cardiovascular death, ischemic stroke, myocardial infarction, systemic embolism, ischemic events requiring urgent revascularization, and major bleeding events within 2 years after randomization.

**Sample size estimates:**

This study will enroll 400 patients, 200 receiving anticoagulant monotherapy and 200 receiving combination therapy. This sample size will provide 90% power (one-sided *p* = 0.025) to detect a risk reduction in outcome events within 2 years, assuming event rates of 13 and 27% for each group, respectively, and a 10% loss to follow-up at a 2.5% significance level with one-sided log-rank tests at an interim analysis and a final analysis.

**Discussion:**

This will be the first study to assess the net clinical benefit of oral anticoagulant monotherapy in ischemic stroke patients with NVAF and atherothrombosis.

**Clinical trial registration:**

https://clinicaltrials.gov/study/NCT03062319, NCT03062319; https://center6.umin.ac.jp/cgi-open-bin/ctr/ctr_view.cgi?recptno=R000029222, UMIN000025392; https://jrct.niph.go.jp/latest-detail/jRCTs051180202, jRCTs051180202.

## 1 Introduction

Non-valvular atrial fibrillation (NVAF) and atherothrombotic disease frequently coexist, particularly in aging populations with shared risk factors such as hypertension and diabetes mellitus (1). Patients with both NVAF and atherothrombotic disease have an elevated risk of ischemic events, including stroke and myocardial infarction, as well as bleeding complications due to antithrombotic therapy. The current guidelines recommend oral anticoagulant therapy (OAC) for the prevention of stroke in patients with non-valvular atrial fibrillation (NVAF) (2, 3) and antiplatelet therapy for patients with non-cardiogenic stroke including large artery atherosclerosis and small vessel disease (4, 5). However, the optimal antithrombotic management for patients with both conditions remains uncertain due to limited evidence.

The combination of OAC and antiplatelet agents is often considered to reduce the risk of recurrent ischemic events (6, 7). However, recent studies have raised concerns regarding the safety and efficacy of this approach. The addition of antiplatelet agents to OAC has been associated with an increased risk of major bleeding (8). The AFIRE trial demonstrated that rivaroxaban monotherapy was non-inferior to combination therapy in terms of efficacy while significantly reducing bleeding risks in patients with NVAF and stable coronary artery disease (9). More recently, the EPIC-CAD trial also demonstrated that edoxaban monotherapy had higher clinical benefit than combination therapy in patients with NVAF and stable coronary artery disease (10). Meta-analyses provide further support for these findings, indicating that combination therapy may not provide a net clinical benefit over anticoagulant monotherapy (11). According to these results, the recent guidelines emphasize the importance of individualized antithrombotic therapy in patients with NVAF and atherothrombotic disease (12).

Furthermore, patients with a history of stroke or transient ischemic attack (TIA) who are receiving antiplatelet therapy are at an even higher risk of major bleeding when treated with OAC (8). Observational studies have demonstrated that the concomitant use of antiplatelet agents with OAC increases the composite outcome of cardiovascular and bleeding events without reducing recurrent stroke in patients with NVAF (13, 14). However, these findings are derived from non-randomized studies, and randomized controlled trials are required to establish the clinical benefit of OAC monotherapy in this context.

The optimal Antithrombotic Therapy in Ischemic Stroke patients with Non-Valvular Atrial Fibrillation and atherothrombosis (ATIS-NVAF) trial is designed to address this evidence gap. This investigator-initiated, prospective, multicenter, randomized control trial aims to evaluate the clinical benefits of OAC monotherapy compared to combination therapy with OAC and antiplatelet agents in patients who have experienced a recent ischemic stroke or TIA and have both NVAF and atherothrombotic disease. The results of this study could have significant implications for clinical practice, potentially informing guideline recommendations and optimizing antithrombotic therapy to improve patient outcomes.

## 2 Methods

### 2.1 Design

This is a protocol for an investigator-initiated, multicenter, prospective, randomized, open-label, parallel-group phase IV study.

### 2.2 Patient population

**Figure 1:**
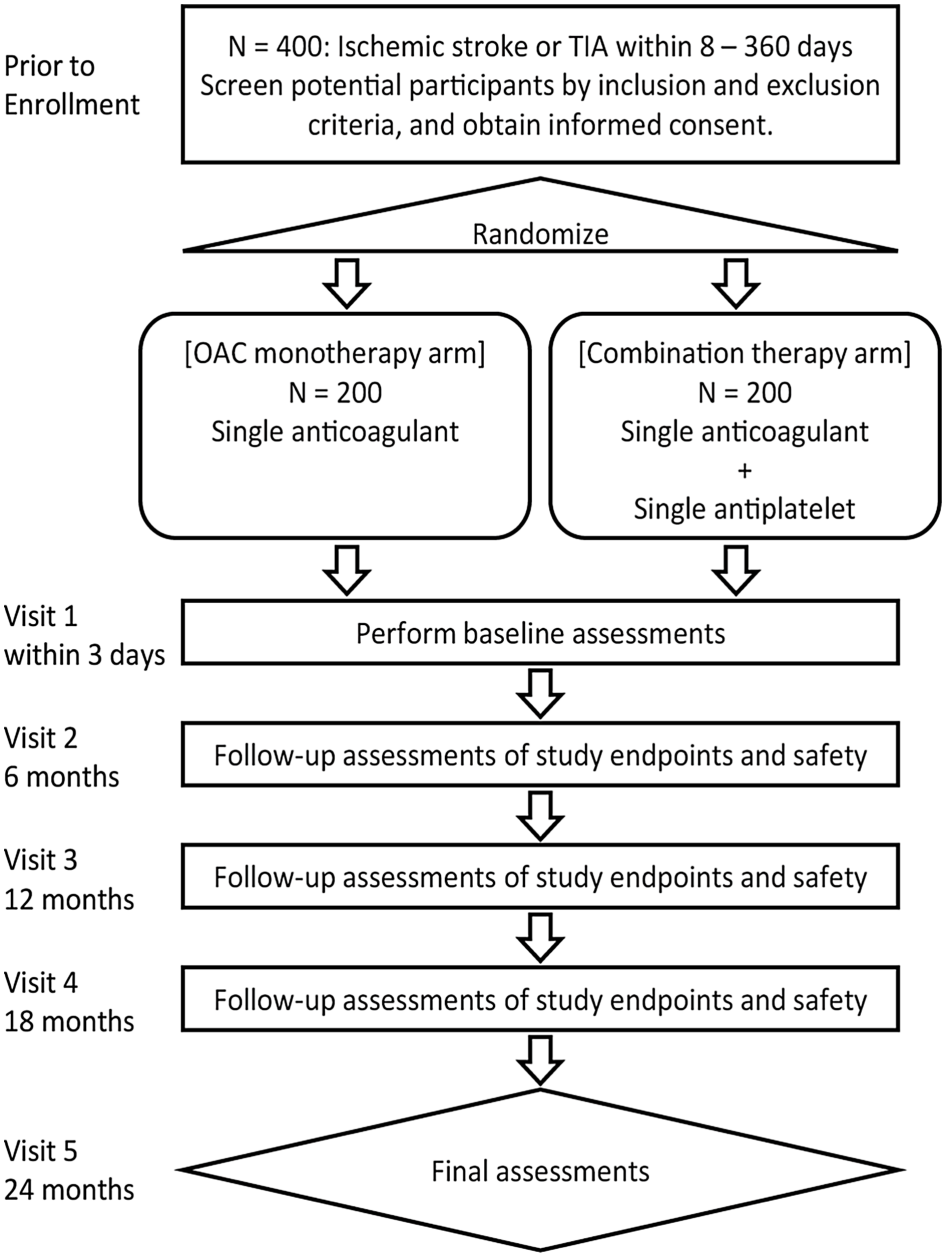
Study design flowchart. OAC, Oral anticoagulant therapy; TIA, Transient ischemic attack.

This trial will be conducted at 41 sites in Japan. The inclusion and exclusion criteria for study enrollment are listed in Table 1. Eligible patients will be identified by their physicians during routine consultations. Figure 1 shows the flowchart of the study design.

**Table 1:** Eligibility criteria.

Inclusion criteria:
Acute ischemic stroke or TIA 8–360 days after symptom onset.
Age: ≥20 years
Patients with non-valvular atrial fibrillation (chronic or paroxysmal) who started or continued taking oral anticoagulants.
Patients with one of the following atherothrombotic diseases:
History of ischemic heart disease (myocardial infarction, angina pectoris, coronary artery bypass graft, or PCI).
History of peripheral artery disease (symptomatic peripheral arterial occlusive disease, lower-extremity bypass surgery/angioplasty/stenting).
Carotid artery stenosis (symptomatic or asymptomatic, ≥50% diameter), a history of carotid artery stenting or carotid endarterectomy.
Intracranial artery stenosis (≥50% stenosis of the diameter of a major intracranial artery: intracranial internal carotid artery, anterior cerebral artery A1 and A2, middle cerebral artery M1 and M2, posterior cerebral artery P1 and P2, vertebral artery, and basilar artery; a history of intracranial stent placement or intracranial bypass surgery).
History of ischemic stroke due to large-artery atherosclerosis or small-vessel occlusion.
Patients with modified Rankin Scale score ≤ 4.
Patients who can take oral medications.
Patients who can complete a follow-up survey.
Provision of written informed consent either directly or by a suitable surrogate.
Exclusion criteria:
History of myocardial infarction or acute coronary syndrome within the past 12 months.
Patients who underwent PCI with drug-eluting stents within the past 12 months or PCI with bare-metal stents within the past 3 months.
Patients who underwent carotid artery, intracranial, or lower-extremity stent placement within the past 3 months.
History of symptomatic intracranial hemorrhage or gastrointestinal bleeding within the past 6 months.
Hemorrhagic diathesis or blood coagulation disorders.
Platelet counts <100,000/mm^3^ at enrollment.
Severe anemia (hemoglobin <7 g/dL)
Severe renal failure (e.g., creatinine clearance ≤15 mL/min) or chronic hemodialysis.
Severe liver dysfunction (Grade B or C of the Child-Pugh classification).
Pregnant women
Active cancer
Expectation of survival <2 years
Anticoagulants or antiplatelets scheduled to be discontinued for >4 weeks during the follow-up period.
Planned revascularization procedure during the follow-up period.
Patients enrolled in other trials.
Patients judged as inappropriate for this study by the investigators.

PCI, Percutaneous coronary intervention; TIA, Transient ischemic attack.

### 2.3 Randomization

All patients who consent to participate and fulfill the eligibility criteria will be randomized to either the OAC monotherapy group or the combination therapy group with a 1:1 allocation according to a computer-generated randomization schedule using the Pocock-Simon minimization method, with adjustment for sex, history of ischemic heart disease, and history of acute ischemic stroke.

The participants will be randomized using the cloud-based electronic data capture service DDworks 21 (Fujitsu Ltd., Tokyo, Japan), an online central randomization service. Allocation concealment will be ensured because the service will not release the randomization code until the patient has been recruited into the trial, which takes place after all baseline measurements have been completed. Clinical events will be assessed by a blinded Independent Central Review Committee. Additionally, all other individuals involved in the study will be blinded to the treatment allocation.

### 2.4 Intervention

Patients will receive either OAC monotherapy or OAC plus antiplatelet therapy at the discretion of the treating physician, in accordance with the protocols outlined in Table 2. The dose of each drug is consistent with the approved dose in Japan. Details of the drugs to be used in both groups are shown in Table 2. Treatment will start within 3 days after randomization and continue for 2 years. If the treating physician needs to change the OAC or antiplatelet drug during the study, the choice of drug may be changed based on the predefined drugs for each group (Table 2). Temporary interruption of study treatment is allowed during invasive procedures such as surgery but cannot exceed 4 weeks. Patients in both treatment groups will be treated according to the relevant guidelines for the prevention of stroke or systemic embolism.

**Table 2:** Allowed anticoagulant and antiplatelet drugs.

Anticoagulants
Select one of the following drugs:
Warfarin
Target INR of 2.0–3.0 if <70 years
Target INR of 1.6–2.6 if ≥70 years
Dabigatran
Full dose: 150 mg BID
Reduced dose: 110 mg BID if CCr <50 mL/min OR age ≥ 70 years OR history of gastrointestinal bleeding OR concomitant P-glycoprotein inhibitors
Rivaroxaban
Full dose: 15 mg OD
Reduced dose: 10 mg OD if CCr <50 mL/min
Apixaban
Full dose: 5 mg BID
Reduced dose: 2.5 mg BID if 2 of 3: age ≥ 80 years OR body weight ≤ 60 kg OR SCr ≥1.5 mg/dL
Edoxaban
Full dose: 60 mg OD
Reduced dose: 30 mg OD if body weight ≤ 60 kg OR CCr <50 mL/min OR concomitant P-glycoprotein inhibitors.
Antiplatelets
Select one of the following drugs:
Aspirin: 75–200 mg OD
Clopidogrel: 50 or 75 mg OD
Prasugrel: 3.75 mg OD
Ticlopidine: 100 mg BID OR 200 mg OD
Cilostazol: 50 or 100 mg BID

BID, Twice daily; CCr, Creatinine clearance; INR, International normalized ratio; OD, Once daily; SCr, Serum creatinine.

### 2.5 Study outcomes

Follow-up visits will be scheduled at 6 ± 2, 12 ± 2, 18 ± 2, and 24 ± 2 months. Each follow-up visit will be conducted by the treating physician. Blood pressure measurements and laboratory examinations will be performed. Patients will be asked about any outcomes or adverse events that occurred before the follow-up visit.

The primary outcome is a composite of ischemic cardiovascular events, including cardiovascular death, ischemic stroke, myocardial infarction, systemic embolism, ischemic events requiring urgent revascularization, and significant bleeding, as defined by the International Society of Thrombosis and Hemostasis (ISTH) criteria (15) within 2 years after randomization.

Secondary outcomes include incidences of the following events within 2 years after randomization: (1) all-cause mortality; (2) ischemic cardiovascular events (cardiovascular death, ischemic stroke, myocardial infarction, systemic embolism, and ischemic events requiring urgent revascularization); (3) all ischemic cardiovascular events (ischemic cardiovascular events, TIA, unstable angina pectoris, and progression of symptomatic peripheral artery disease); and (4) ischemic stroke; and (5) myocardial infarction and cardiovascular death.

Safety outcomes include incidences of the following events within 2 years after randomization: (1) ISTH major bleeding; (2) ISTH major bleeding and clinically relevant non-major bleeding; (3) intracranial hemorrhage.

### 2.6 Sample size estimates

The sample size was calculated based on the primary hypothesis and the results of our previous study. A retrospective study of 277 patients with NVAF and atherothrombotic disease demonstrated that the combination therapy group had a higher incidence of ischemic cardiovascular events and major bleeding than the OAC monotherapy group (33% vs. 19%/2 years, log-rank *p* = 0.04; unpublished data). Based on this result, we calculated that a maximum total sample size of 370 (equally-sized groups and the maximum number of events is 74) would be required to provide a power of 90% to detect a risk reduction in outcome events within 2 years, assuming lower event rates of 13 and 27% for each group, respectively, at an overall significance level of 2.5% with one-sided log-rank tests at an interim analysis and a final analysis (EAST® version 6.4, Cytel, Boston, MA, United States). An interim analysis was planned to assess the efficacy and futility when half the required number of events was observed. The critical values for the analyses were determined based on the Lan-DeMets error-spending method using an O’Brien-Fleming-type function with a non-binding boundary for futility. With a proposed 10% dropout rate, 400 participants will be recruited for this study.

### 2.7 Statistical analyses

Analyses will be performed based on the intention-to-treat (ITT) principle. The data will be summarized using descriptive statistics. For the primary endpoint, survival curves will be estimated using the Kaplan–Meier method and compared between the two groups using the log-rank test. Hazard ratios with 95% confidence intervals will be calculated using a proportional hazards model. A per-protocol analysis will be performed to support the conclusions derived from the ITT-based analysis. Additionally, homogeneity of the treatment effects will be exploratively evaluated with the following subgroups: age (<80 vs. ≥80), sex, anticoagulant drug (warfarin vs. direct OACs), CHADS2 score (≤3 vs. 4 vs. ≥ 5), body weight (<60 kg vs. ≥60 kg), creatinine clearance (<30 mL/min vs. 30 ~ 50 mL/min vs. ≥50 mL/min), history of ischemic stroke and major bleeding, and prevalence of carotid or intracranial artery stenosis, hypertension, diabetes mellitus, dyslipidemia, and congestive heart failure.

Secondary endpoints will be analyzed in the same manner as the primary endpoints, except for the calculation of *p* values. Safety data will be analyzed descriptively for the treated set, which consists of all randomized patients who received at least one study treatment. The detailed plan for the interim and final analyses will be prespecified in the statistical analysis plan and finalized before the database lock. All statistical analyses will be conducted at the data center in the National Cerebral and Cardiovascular Center.

### 2.8 Safety and data monitoring body

Data management, statistical analysis, and monitoring will be performed by the Department of Data Science at the National Cerebral and Cardiovascular Center in Suita, Japan. An independent Data Safety Monitoring Board (DSMB), comprising two stroke neurologists and a biostatistician, will be created to monitor the safety and efficacy of the study. The DSMB will recommend trial discontinuation/continuation or protocol amendment based on annual reviews of patient accrual, the incidence of adverse events, and interim efficacy analyses comparing the two treatment arms.

### 2.9 Study organization and funding

This study is partially supported by the Japan Thrombosis Investigator Initiated Research Program (JRISTA), funded by Bristol-Myers Squibb (BMS) and Pfizer. Bristol-Myers Squibb (BMS) and Pfizer were not involved in the planning, data management, analysis, or discussion of the study results. The final trial protocol was prepared by the Protocol Committee. An independent data-monitoring committee will adjudicate new outcomes at regular intervals. The committee receives outcome information and is blinded to patient identifiers and treatment allocation.

## 3 Discussion

The ATIS-NVAF is the first randomized trial to evaluate the safety and efficacy of OAC monotherapy in a population at risk for both cardiogenic embolism and atherothrombotic disease. The data obtained from this trial will be crucial for identifying optimal medical treatment for these patients.

The study design focuses on comparing OAC monotherapy with combination therapy of OAC and antiplatelet agents, without imposing any specific antiplatelet or OAC regimens, to reflect real-world situations. Since the safety profile of direct OACs in patients with end-stage renal failure has not been established (16), warfarin or warfarin and antiplatelet therapy are often used in clinical practice for patients with cardioembolic stroke, renal failure, and ischemic heart disease. This study allows the use of warfarin in the anticoagulation group to explore optimal antithrombotic treatment strategies for high-risk patients.

Recent evidence highlights that inflammation and endothelial dysfunction are key contributors to both atrial fibrillation and atherosclerosis (17, 18). In atrial fibrillation, endothelial injury promotes thrombogenesis (17). Similarly in atherosclerosis, endothelial dysfunction increases the risk of thrombotic events (19). These shared mechanisms emphasize the necessity for optimized antithrombotic therapy in patients with both conditions.

Although the ATIS-NVAF trial is designed to reflect real-world clinical practice by allowing flexibility in treatment selection, this approach introduces certain limitations. First, the open-label design of the study may introduce bias, as both patients and physicians are aware of the assigned treatments. This awareness may potentially influence patient adherence, reporting of outcomes, and clinical decision-making by physicians. Second, the selection of specific OACs and antiplatelet agents, as well as their dosage, is left to the discretion of the treating physicians within the predefined options. This variability may lead to heterogeneity in the interventions, which could affect the internal validity of the study. These limitations should be considered when interpreting the results of the ATIS-NVAF trial. Despite these challenges, the pragmatic design enhances the external validity and applicability of the findings to routine clinical practice, which is a significant strength of the study.

In conclusion, this study will be the first to assess the net clinical benefit of OAC monotherapy in patients with ischemic stroke with NVAF and atherothrombosis.
